# The brain in acute on chronic liver failure

**DOI:** 10.1007/s11011-014-9553-0

**Published:** 2014-05-20

**Authors:** Gavin Wright, Yalda Sharifi, Maria Jover-Cobos, Rajiv Jalan

**Affiliations:** 1 Institute for Liver and Digestive Health, Liver Failure Group, UCL Institute of Hepatology, The Royal Free Hospital, Upper Third UCL Medical School, Pond Street, London, NW3 2PF UK; 2 Basildon & Thurrock University Hospitals NHS Foundation Trust Nethermayne, Essex, SS16 5NL Basildon UK

**Keywords:** ACLF brain, Hepatic Encephalopathy, Hyperammonemia

## Abstract

Acute-on-chronic liver failure (ACLF) is a newly defined clinical entity with significant morbidity and mortality (~40–90 % at 1 year dependent on need for organ support at presentation). It defines a presentation with acute severe liver injury, often with multiorgan dysfunction, on a background of previously known or unknown cirrhosis. In its severest form, it is almost indistinguishable from acute liver failure, as similarly in around 5 % may rapidly progress to intracranial hypertension and cerebral oedema culminating in coma and/or death. Our understanding of such cerebral sequelae is currently limited to clinical observation, though our knowledge base is rapidly expanding since recent consensus clinical definition and guidance. Moreover, there are now animal models of ACLF and imaging modalities to better characterize events in the brain that occur with ACLF. However, as yet there has been little in the way of interventional study of this condition which are much needed. In this review we dissect existing clinical and experimental data to better characterise the manifestations of ACLF on the brain and allow for the development of targeted therapy as currently the plethora of existing interventions were designed to treat either the effects of cirrhosis or acute liver injury independently.

## Introduction

Acute-on-chronic liver failure (ACLF) is a new clinical entity with significant morbidity and mortality. In its severest form it is almost indistinguishable from acute liver failure, aside from background cirrhosis, as similarly may rapidly progress to intracranial hypertension and cerebral oedema culminating in coma and/or death. This happens in around 5 %, but our understanding of such cerebral sequelae is limited as little clinical or animal model data exists to-date. In this review we dissect existing clinical and experimental data to better characterise the manifestations of ACLF on the brain and allow for the development of targeted therapy as currently the plethora of existing interventions were designed to treat either the effects of cirrhosis or acute liver injury independently.

## Types of liver failure

Hepatic encephalopathy (HE) is a neuropsychiatric syndrome that may develop with advancing liver insufficiency and helps define the differing clinical syndromes of liver failure. Acute liver failure (ALF) is defined by the presence of HE within 8-weeks of the initial liver insult, in the absence of chronic liver disease (e.g. cirrhosis). In its most advanced stages it is associated with intracranial hypertension and possible death from brain herniation (Clemmesen et al. 1999). In cirrhosis, HE typically occurs insidiously with wide ranging neuropsychiatric disturbances (e.g. psychomotor dysfunction, impaired memory, decreased reaction time, diminished attention, sensory abnormalities and poor concentration). However, there is a growing number of patients with cirrhosis presenting more acutely in a manner better represented by ALF, with acute end-organ dysfunction(s) requiring support. This new clinical entity - termed ‘*acute-on-chronic liver failure*’ (ACLF), defines acute deterioration in patients with cirrhosis that triggers cerebral and clinical changes indistinct from ALF.

One of the first attempts to define ‘acute-on-chronic liver failure’ (ACLF) was by the London group in 2002 who suggested that ACLF encompasses the development of cerebral and clinical changes indistinct from ALF following acute liver injury on the background of chronic liver disease (Sen et al. 2002). However, as a new clinical entity, consensus over what defines ACLF remains both topical and debated. The most accepted ACLF definition to date have been set out by:

1. the Asia-Pacific Association for the Study of Liver Disease (APASL)—‘Acute hepatic insult manifesting as jaundice and coagulopathy, complicated within 4 weeks by ascites and/or encephalopathy in a patient with previously diagnosed or undiagnosed chronic liver disease’ (Sarin et al. 2009).
2. EASL-AASLD single topic symposium—‘Acute deterioration of pre-existing chronic liver disease, usually related to a precipitating event and associated with increased mortality at 3 months due to multi-system organ failure’ (Olson et al. 2011).

## ACLF is distinct

On review of existing ACLF data, there is marked variance in hard clinical outcomes such as ICU mortality (35 to 89 %) and in-hospital mortality (43 to 88 %). This disparity may directly reflect the definition used and the resultant therapeutic and management practices applied. Given differing morbidity and mortality outcomes there is a fundamental need to provide a clear distinction between ‘true’ ACLF and less severe decompensated chronic liver disease. In ACLF patients requiring intensive care unit (ICU) support (Olson et al. 2011), reported 53 % ICU mortality (and mean 14-day hospitalization), whereas others suggest a mortality as high as 89 % (Levesque et al. 2013). Higher mortality is usually indicated by the extent of organ support required at admission, with Levesque et al. reporting 89 % 1-year mortality in those requiring mechanical ventilation. In support of earlier ACLF literature, prognostic severity scores, need for other organ support therapy, infection and total bilirubin at ICU admission were associated with high rates of ICU mortality. They also report that of the 34 % discharged from ICU, 1-year survival was still only 32 %; with total bilirubin <64.5 micromol/L and length of ventilation >9 days were independent indicators of poor prognosis (Levesque et al. 2013).

Given increasing shortage of ICU capacity, such mortality risk stratifiers could temper our enthusiasm to provide early support for ACLF patients if certain indicators are unfavourable at presentation. However, morbidity and mortality has improved significantly for this at risk group due to early organ support and/or restorative intervention and that targeting the earlier identification of this at-risk group at hospital admission with early high-intensity care may be more beneficial in terms of morbidity, mortality and health-economic considerations. As such development of ACLF care-bundles (covering the first 48–72-hours of admission) to aid access and application of best-practice and specialist care is warranted.

## The brain in ACLF

### Clinical description of the brain in ACLF

As with ALF, HE is a common manifestation of ACLF (Jalan et al. 2002) and from the pathophysiological perspective similarly may progress to intracranial hypertension and cerebral oedema (Crippin et al. 1992; Jalan et al. 1997; Donovan et al. 1998). In respect to the changes within the brain in ACLF, the first acknowledgement of this hitherto unknown phenotype as distinct from decompensated cirrhosis and ALF was reported by Jalan et al. 1997; (Jalan et al. 1997) They described the previously unreported phenomenon of acute intracranial hypertension and oedema (c.f. ALF) in four patients with cirrhosis following emergency transjugular intrahepatic porto-systemic shunt (TIPS) for variceal haemorrhage, associated with a marked deterioration in liver function tests. The current explanation for this post-TIPS effect relates to increased systemic microbial load secondary to redirected portal blood into the systemic circulation with therapeutic shunting. Such changes are associated with increased nitric oxide (NO) production possibly through an iNOS dependent mechanism with severe pathophysiological effects leading to circulatory failure in the critically ill cirrhotic patient (Jalan et al. 2011).

Donovan et al. later described their experience of 12 patients presenting with acutely decompensated cirrhosis with clinical and radiological evidence of raised ICP and brain oedema. They successfully treated two patients with transplantation which resulted in clinical neurological resolution. This importantly shows a reversible component to the ACLF brain, further indicating a potential therapeutic window for intervention to target cerebral sequelae of clinical ACLF and hopefully improve morbidity and mortality in this hitherto often fatal clinical presentation (Donovan et al. 1998).

Cordoba et al. expanded on our clinical understanding of the cerebral effects of ACLF by showing in similarly cerebrally obtunded ACLF patients demonstrating resolution of cerebral oedema and amelioration of cerebral haemodynamics on MRI, again highlighting the potential for reversibility, similar to that seen with ALF, despite the background cirrhotic brain phenotype (García Martínez et al. 2010). Moreover, in a recent large comparative clinical study of consecutive decompensated cirrhosis (*n* = 138) versus ACLF (*n* = 301) patients, from the CANONIC Study database, Cordoba et al. elegantly demonstrate that HE associated with ACLF predominately occurs in younger often alcoholic patients, associated with more severe liver failure and systemic inflammatory response syndrome (SIRS) with extremely poor prognosis with age, bilirubin, INR, creatinine, sodium and HE grade, independent risk factors for mortality. Conversely decompensated disease occurred more frequently in older usually inactive drinkers, without severe liver failure or SIRS and often associated with diuretic use (Cordoba et al. 2014).

There has often been debate as to how frequently brain oedema and raised ICP in ACLF occurs, but new data from Joshi et.al., indicates that less than 1 in 20 (~5 %) of such ACLF patients formally progress to such an advanced stage (Joshi et al. 2013). This is likely to be an underestimation as the patients were not monitored and given that patients with cirrhosis have some degree of cerebral atrophy, any cerebral oedema will not result in obvious clinical manifestations of increased intracranial pressure.

Other local and systemic factors have been implicated in the pathophysiology and development of this neurological syndrome. Changes in ammonia levels and inflammatory status, along with changes in cerebral haemodynamics make up our current paradigm for HE pathogenesis but their relative contribution to progression of HE in ACLF as opposed to that seen with ALF and cirrhosis has not been clearly defined (Shawcross et al. 2011). The importance of infection/SIRS in precipitating HE is well described in ALF and cirrhosis, but not so in ACLF. Shawcross et.al, have recently demonstrated the somewhat expected correlation between infection/SIRS and advanced HE (grades 3–4) in cirrhotic patients admitted to ITU primarily for encephalopathy (Shawcross et al. 2011). This study also discusses the relative role of ammonia in development of HE in ACLF, as plasma ammonia was not clearly associated with advancing HE but most patients were hyperammonaemic.

### Evidence of brain oedema in ACLF

As there is no human histopathological support for the clinical findings in ACLF, there is a reliance on interpretation of animal data in models of ACLF along with the translation of clinical data from ALF and cirrhosis patients where similarities exist. Precipitants of advanced HE in ACLF are the same triggers for general decompensated cirrhosis and it remains unclear as to why in any one patient a neurological decline to intracranial hypertension and cerebral oedema ensues. One can postulate that a shift in neurophysiology may be triggered by either 1) a more significant pathological challenge (e.g. microbial inoculate dose, massive GI bleed and/or SIRS etc.), 2) background liver capacity and/or 3) local brain neurophysiology and anatomy. This latter point has been suggested in data from rat models of ACLF around a concept of ‘priming’, which will be discussed later.

#### Pathogenic synergism ammonia and inflammation

Ammonia has been shown to correlate with the severity of HE in cirrhotic patients (Ong et al. 2003), and predict brain herniation in patients with ALF (Clemmesen et al. 1999). Pathophysiologically, astrocyte metabolism of ammonia produces accumulation of osmotically active glutamine to produce brain oedema and intracranial hypertension (Haussinger et al. 1994; Cordoba et al. 1996; Tofteng et al. 2006). In patients with ALF, SIRS (often secondary to infection) correlates with advancing stages of HE and intracranial hypertension (Rolando et al. 2000; Vaquero et al. 2003). We have previously reported a correlation between intracranial hypertension and both circulating and brain levels of pro-inflammatory cytokines in ALF patients (Jalan et al. 2004; Wright et al. 2007a). In patients with cirrhosis and SIRS/infection, induced hyperammonaemia worsened neuropsychological function; this suggests pathogenic synergy between hyperammonaemia and inflammation (Shawcross et al. 2004). However, from these clinical studies it is not clear whether it is the background state of the brain in cirrhosis or the associated hyperammonaemia that predisposes to the effects of the superimposed inflammation. In a histological study of a rodent model of ACLF, Wright et al., observed that acute severe microbial (Lipopolysaccharide; LPS) challenge to bile-duct ligated (BDL) rat model of cirrhosis induced marked increase in brain water (with astrocyte and associated perivascular oedema), mimicking ACLF. However, more severe LPS-induced brain oedema was evident in naïve non-cirrhotic rats with induced hyperammonaemia. Interestingly, only LPS challenged BDL rats reached pre-coma stages at 3-hours despite more severe brain oedema in LPS-challenged hyperammonaemic naïve rats. This decoupling of increased brain water and preserved mental state in all rats except LPS-treated cirrhotic rats suggests that factors in addition to inflammation, hyperammonaemia and resultant brain swelling contribute to the effects on consciousness found in HE.

#### Blood brain barrier integrity (Cytotoxic versus Vasogenic Oedema)

Histological analysis indicates that increases in brain water are indistinguishable from the well-documented brain oedema seen in hyperammonaemic ALF models. However, distinct from these ALF models where the blood–brain barrier (BBB) was observed to be compromised (Traber et al. 1986; Kato et al. 1989) the anatomical integrity of the BBB is maintained with retention of the ionic tracer lanthanum nitrate (Molecular weight 433 kDa) on electron microscopy. This difference may suggest a specific phenomenon of cytotoxic oedema in cirrhotic rats compared with a combined cytotoxic and vasogenic oedema in ALF. Wright et al., also looked at possible associated pathophysiological changes in the brain physiology. The astrocytic oedema in the non-cirrhotic hyperammonaemic rats was expectedly associated with increased glutamine, reduction in *myo*-inositol and a marked increase in the glutamine/myo-inositol ratio in keeping with the ammonia-glutamine-brain water hypothesis. However, the mild astrocytic oedema seen in the saline-treated BDL rats was markedly less despite similar degrees of plasma and brain ammonia levels as seen with the LPS-administered BDL group. The mechanisms underpinning astrocytic swelling in cirrhotic rats are therefore unclear and possibly influenced by the effects of inflammation. Yet worsening oedema following LPS-challenge in this study did not directly correlate with plasma and brain ammonia levels or brain ammonia metabolism. These experimental data may provide some insight into why ammonia levels show no direct correlation with advanced HE (grade 4) and outcomes in the clinical ACLF study by Joshi et.al., reported earlier (Joshi et al. 2013).

#### Systemic versus brain inflammatory processes

Uniquely this study also tied the marked SIRS (peripheral trigger) to brain inflammatory responses with increased brain tissue TNF-α confirming our previous observation that a systemic inflammatory response may initiate brain inflammation during liver failure despite retained anatomical barrier integrity. In BDL rats high nitric oxide levels and hyperammonaemia were further associated with ammonia-induced nitrosation of astrocytic proteins (e.g. protein tyrosine nitration -PTN). Although inflammation may act synergistically with ammonia, LPS can itself lead to nitrosation of proteins in the brain (Lee et al. 2005). This may indicate that in cirrhosis, the existent background hyperammonaemia and low-grade inflammatory cerebral milieu (Jover et al. 2006) may ‘prime’ the animal to the effect of subsequent endotoxaemic/inflammatory insult. This concept of pathogenic synergy fits with the clinical data (Shawcross et al. 2004).

#### Role of oxidative stress

Whether due to local cellular (e.g. astroglial) dysfunction, or peripherally (e.g. other end-organ dysfunction and/or circulating cells like neutrophils), resultant free-radical formation and/or oxidative stress, the cellular dysfunction caused by imbalanced creation, breakdown or inhibition of reactive oxygen species (ROS) and/or antioxidants, appear equally pivotal to ammonia-induced neurotoxicity. Data from both animal and human studies also suggest potential pathogenic synergy between systemic oxidative stress, and ammonia in HE, with rising ammonia levels associated with progressive oxidative stress and also free-radical production mediated by NMDA-receptor activation and ammonia-induced mitochondrial dysfunction (Marcaida et al. 1992). This could also be a source of ROS such as peroxynitrite (OONO^•^), itself mediating NO-induced blood brain barrier damage and astocyte dysfunction (potentially through PTN), which has been shown in models of hyperammonaemic acute or chronic liver injury and in cell culture (Bosoi et al. 2012; Jayakumar et al. 2006; Norenberg et al. 2005; Schliess et al. 2004; Rao et al. 2001); we have similarly shown this in a LPS-challenged BDL model of ACLF (Wright et al. 2007b). Oxidative stress occurs more robustly with the acute and significant hyperammonaemic insult of ALF and ACLF (Wright et al. 2007a, b), as opposed to the more insidious, though no less profound hyperammonaemia of CLD. This is an interesting observation which again points to the importance of the chronicity of injury rather than absolute level of hyperammonaemia in the clinical phenotype of HE. The likely importance of oxidative stress in HE pathogenesis also provides a the rationale for using antioxidants (e.g. N-Acetylcysteine) to ameliorate brain sequelae associated with advanced liver disease, which has proven beneficial in animal models of HE (Guerrini 1994).

#### Role of cerebral haemodynamics

Cerebral blood flow (CBF) is integral to the HE pathogenesis and likely directly linked to alterations in both ammonia and inflammation as they independently influence cerebral haemodynamics (discussed later). As already discussed, they appear to act synergistically, which has even been shown to influence CBF in non-cirrhotic rat models (Pedersen et al. 2007). Development of cirrhosis is known to engender a progressively reduced CBF (cerebral oligaemia); in contrast ALF is associated to large increases in CBF (cerebral hyperaemia) and eventually ICP, brain oedema, coma and/or death from brain herniation. Paradoxically CBF may increase significantly in ACLF to mimic ALF. In a seminal study of cirrhotic patients receiving TIPS for either GI bleeding or ascites, TIPS-induced endotoxaemia led to an increase in the rate of production of nitric oxide, which was associated with endothelial dysfunction and an increase in CBF (Jalan et al. 2011). However, insertion of TIPS in relatively stable cirrhotics in the absence of HE had no effect on CBF, indicating the likely pathophysiologically distinct nature of ACLF (Iversen et al. 2011). These observation also support the hypothesis that multiple hits and brain swelling is central to the brain manifestations of ACLF.

#### Role of imaging techniques

Given the distinct lack of histological evidence from patients with ACLF, neuroimaging modalities allow for non-invasive assessment of brain alterations in liver failure to better establish the pathogenic mechanisms involved. As the sensitivity of MRI machines improve, it is likely in the not so distant future, this technique will provide a better understanding of HE mechanisms that may prove useful for early diagnosis, local therapy design and monitoring of cerebral complications of liver failure (Chavarria and Cordoba 2013). Nath et al., in a smaller imaging study (using diffusion tensor imaging (DTI) metrics) of 23 ACLF patients compared to healthy controls, demonstrated both intracellular and extracellular brain oedema (Nath et al. 2008). The same group further compared changes in brain osmolytes with ALF, ACLF and stable cirrhosis versus healthy controls (*n* = 10/group) using *in vivo* ^1^H magnetic resonance spectroscopy (Verma et al. 2008) demonstrating lower osmotically active myo-inositol and glutamate/glutamine levels within the brain of ACLF patients. If the astrocyte brain water-glutamine hypothesis is true, this may suggest that the extent of brain oedema in ACLF is less profound than evident with ALF, a concept consistent with the change from vasogenic to cytotoxic with advanced HE grade (e.g. 3–4) in ALF. Recently however, brain lactate, both a brain osmolyte and one of the most important brain energy sources, has been implicated in progression of HE and cerebral oedema in rat models, with rising levels associated with advance stages and a potential therapeutic target (Bosoi et al. 2013). A rise in cerebral lactate may occur due to 1) hyperlactataemia related to compromised end-organ circulation which then crosses the BBB, 2) increased glycolysis due to energy failure or impairment and/or (3) increased lactate production/release or decreased lactate utilization/uptake (Rose 2010). Regardless, aiming to reduce brain lactate by inhibiting lactate synthesis is not without concern as lactate cannot be replaced by glucose as an energy source. Any resultant lack of energy sources could lead to a worsening of the functionality of the brain, inducing worsening of the motor and memory deficits (Oria and Jalan 2013). It therefore remains likely that the increase in brain lactate is a consequence of pathophysiological mechanisms rather than its cause.

### Animal models of ACLF

To-date there are limited animal models of ACLF. This is not unsurprising given that the clinical syndrome has only recently been established and still under a dynamic process of characterization. However, what is common to all reported models of ACLF is the need to initially induce chronic hepatic fibrosis/cirrhosis before induction of failure with developing pathophysiological changes reflecting the typical clinical sequelae. As expressed there has been little advances in recent years to suggest the establishment of newer models of ACLF (Belanger and Butterworth 2005). The most validated ACLF models are:

*D-Galactosamine/LPS-induced inflammatory liver injury model*: This ACLF model was first characterised by Liu and colleagues (Liu et al. 2007) and since utilized in a number of notable studies (Kuhla et al. 2009; Wang et al. 2012). It involves the induction of immune liver fibrosis in rats by multi-point 0.5 ml subcutaneous injections of human serum albumin (HSA), diluted to a concentration of 8 g/L with physiological saline (emulsified by an equal amount of incomplete Freund’s adjuvant). There is a 14-day interval between the first and second injection and 10-day interval between third and fourth. Then, a further 4 mg HSA was injected into rat tail vein twice a week for 6 weeks. Following induced immune liver fibrosis the rats are injected with intraperitoneal D-galactosamine (400 mg/kg) combined with LPS (100 μg/kg) to induce secondary ALF on a background of chronic liver injury.

#### BDL/LPS-induced ACLF model

This model has been used for some time (Harry et al. 1999) and more formally characterized as a model of ACLF by Wright and colleagues (Wright et al. 2007a, b) with special attention to the brain sequelae of this this condition. It involves the induction of secondary biliary fibrosis/cirrhosis by operative BDL, performed on rats under anaesthesia (intravenous diazepam (1 mg/kg), followed by intramuscular Hypnorm® (150 μl/kg)). It involves midline laporotomy and identification of the bile duct with ‘triple’ ligation and then isolation and severance of the bile duct. After post-operative recovery the BDL rats are kept under regulated and controlled conditions with free access to food and water and development of secondary biliary cirrhosis over the following 4–6 weeks with clear evidence of decompensation (e.g. jaundice, ascites). Prior to termination the BDL rats are administered LPS (1 mg/kg) to induce superimposed acute liver injury/failure.

## Multicellular interactions, synergy, priming and immune adaptation

Given the involvement of both systemic and regional end-organ responses, inflammation, ammonia (and amino-acid metabolism) and cerebral haemodynamics, it is important to consider how cellular interactions within the brain allow for these pathophysiological observations.

Ammonia is likely to act as the priming stimulus on the background of which inflammation may produce HE. This concept is supported by interventional studies, a good example of this is the lowering of circulating ammonia with the novel therapy ornithine phenylacetate (OP), which protected against the brain swelling associated with liver failure (Davies et al. 2009), Ammonia is detoxified by the astrocytes in the brain and swell during hyperammonaemia due to the osmotic effect of glutamine (Haussinger et al. 2000). Astrocytes form an integral component of the blood–brain barrier and regulate cerebral blood flow through an arachidonic acid dependent pathway (Takano et al. 2006). This observation directly links a possible mechanism that may underlie the synergy between ammonia and inflammation in which astrocytes are the critical cells. Hyperammonaemia may therefore activate astrocytes ‘unlocking’ the blood–brain barrier (Jalan et al. 2007), making them susceptible to endotoxaemia through a Cyclo-oxygensase (COX)-dependent mechanism. There is also newer evidence proposing a role for microglia (Wright et al. 2013) and pericytes in the regulation of cerebral haemodynamics via their recently reported regulatory role on capillary vascular tone (Peppiatt et al. 2006) but their role in ACLF is still unclear.

## Approaches to prevention and treatment

Ammonia, inflammation, modulation of CBF autoregulation and the precipitants of acute liver injury are central to the cerebral sequelae of ACLF and therefore, important therapeutic targets in the management paradigm. Despite numerous therapeutic approaches to the wide spectrum of neuropsychiatric presentations with liver failure, there is a need for more interventional therapies for those patients with ACLF, as with ALF. Therapies are therefore focused on this mechanisms underpinning this pathogenic mechanisms outlined above. Modulation of ammoniagenesis and inflammatory processes, without compromising global brain homeostasis or inducing systemic complications, therefore remains the key to defining new therapeutic strategies for HE.

**Fig. 1:**
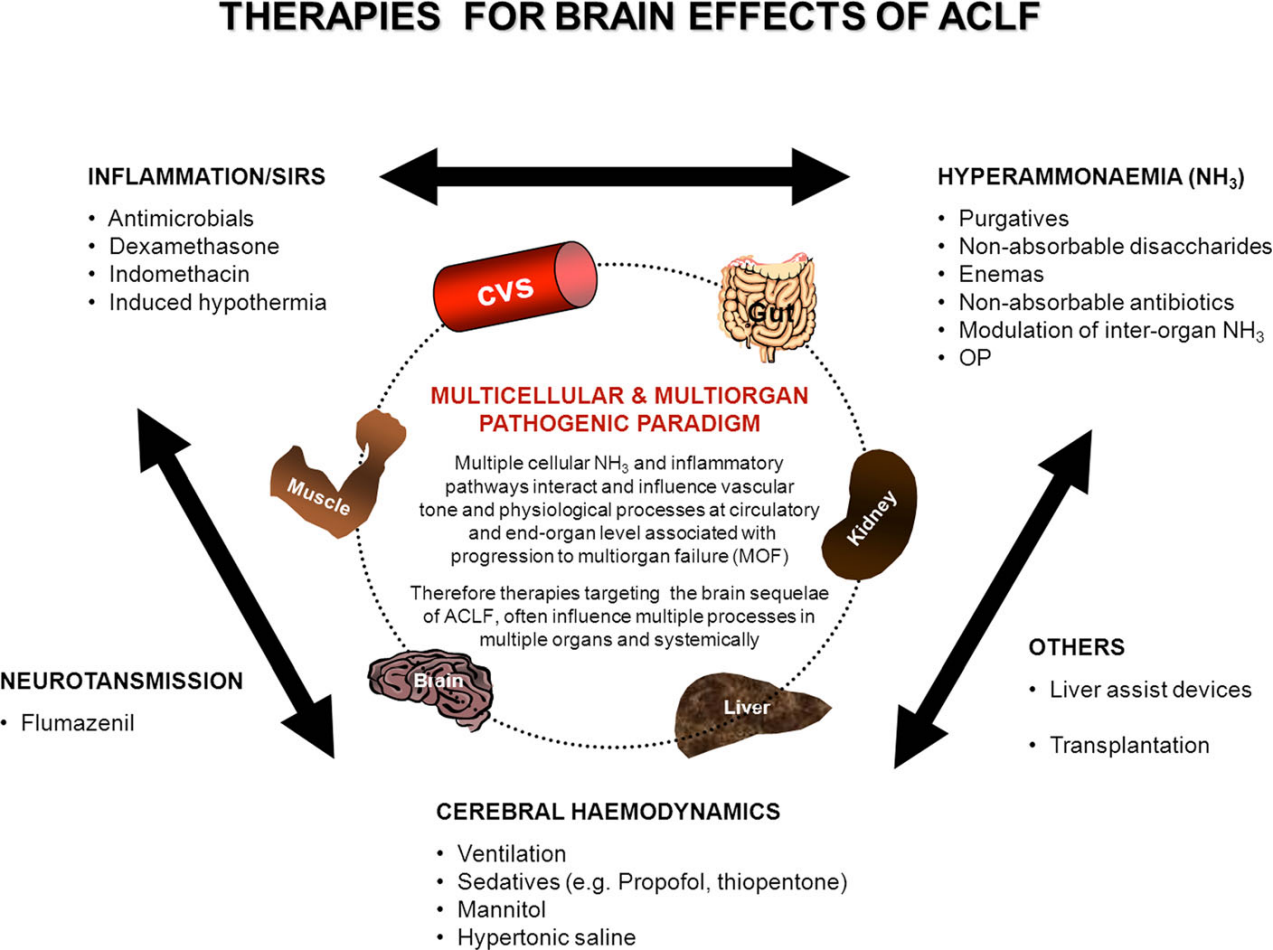
Target Therapies for brain effects of ACLF

In ACLF that progresses to cerebral oedema with intracranial hypertension and resultant coma, the need to treat the multi-organ effects of liver failure are inseparable from treating its cerebral effects. Early ventilation, intensive care unit admission and judicious use of available therapies have led to a significant decline in deaths as a result of cerebral sequelae. Treating precipitating factors is as crucial as directly aiding liver recovery by prompt and specific treatment of the cause of acute liver injury. Though ACLF may be triggered by such uncommon events as sedatives or tranquilizers, vascular occlusion (hepatic vein or portal vein thrombosis) and hepatocellular carcinoma transformation, it is paramount to manage the more common precipitants such as constipation, electrolyte and acid–base imbalance, infection, gastrointestinal bleeding and portosystemic shunts. However, general management of true ACLF requires a multi-organ approach, especially for those in need of formal organ support, given the high mortality rates. There have been no formal clinical ACLF studies directly assessing therapies targeting cerebral effects but what is understood is the applied benefit of ALF therapies, which principally influence cerebral haemodynamics to limit ICP and brain oedema. Figure 1

summarises some of the strategies that need to be assessed in ACLF patients.

### General

#### Ventilation

Early airway maintenance and intubation prevents high carbon dioxide tension and hypoxia limiting cerebral hyperaemia (Ede et al. 1986). Good airways protection limits risk of aspiration, defective gas exchange and infection. Propofol is often preferred as the sedative because it reduces ICP (though may cause hypotension) (Wijdicks and Nyberg 2002), and due to non-hepatic metabolism does not accumulate.

#### Circulatory support and fluid management

Like ALF, ACLF is a hyperdynamic state with high cardiac output, low mean arterial pressure (MAP), and low systemic vascular resistance. Moreover given background cirrhosis there is already generalized vasodilatation, profound neurohormonal activation and resultant vasoconstricted regional vascular beds (Jalan 2005). MAP should be maintained at a level to keep CPP between 50 and 65 mmHg (Davies et al. 1994). Ensuing Multi-organ failure (MOF) often necessitates inotropes; in those refractory to inotropes a short synacthen test on ITU admission guides steroid use as adrenal insufficiency (common in cirrhosis) (Harry et al. 2002).

#### Electrolyte imbalance

Hyponatraemia ≤125 mmol/L may precipitate cerebral oedema and is a contraindication for orthotopic liver transplant (OLT) (Jalan et al. 1997; Cordoba et al. 1998). In ALF, induced hypernatraemia may improve ICP and reduce inotropic requirements (Murphy et al. 2004), though potential benefit has not been formally assessed in ACLF patients.

#### Antibiotic/antimicrobial agent

Given the incidence of infection/SIRS related neurological decline, empirical broad-spectrum antibiotics/antifungal is a requirement and should be targeted once an organism identified (Rolando et al. 1990, 2000).

#### Glycaemic control

Disturbed glycaemic and lipid control is common in progressive liver disease and worsened by the stress response in ACLF that could provoke neuroglycopenia and brain oedema. Tight glycaemic control using insulin reduces oxidative stress (which triggers insulin resistance), limits mitochondrial liver damage, and improves endothelial nitric oxide activation to optimize blood flow, limiting tissue injury, and improve outcomes (Langouche et al. 2005; Houstis et al. 2006).

### Specific

In ALF there are many therapies utilized to lower intracranial hypertension and oedema. However such therapies (e.g. mannitol, hypothermia, hypernatraemia, anti-epileptics and indomethacin) currently have no evidence in ACLF and require evaluation in specific ACLF clinical trials. In respect to ammonia-lowering therapy, although disaccharides are useful in ALF, the data of its usefulness in ACLF patients is lacking. Otherwise none of the ammonia-lowering therapies have been trialled in this condition although as discussed previously ornithine phenylacetate may have a role but will need to be proven in appropriate clinical trials.

#### Albumin dialysis using molecular adsorbents recirculating system

The extracorporeal device that has had the most clinical evaluation is the ‘Molecular Adsorbent Recirculating System (MARS)’ that provides counter-current dialysis against albumin and bicarbonate circuits. Albumin dialysis with MARS decreases retained substances and improves haemodynamics and HE. In a randomised clinical trial of MARS in ACLF patients performed in the US, there was a significant benefit of MARS in reducing the time to wake up and effectiveness. In another multinational, multicentre trial of 189 ACLF patients randomized to MARS (*n* = 95) or standard therapy (SMT) (*n* = 94), there was no significant beneficial effect of MARS on 28-day survival (~60 %). However, MARS had an acceptable safety profile, significant dialysis effect and non-significantly improved advanced HE (3–4) grades (Bañares et al. 2013).

## Conclusion

The brain sequelae of clinical ACLF are often profound and associated with significant morbidity and mortality. As yet there has been little study in either humans or animal models of ACLF that allow us to better elucidate the histopathophysiological characteristics and define the syndrome. However, there is some understanding of managing the clinical presentation using a plethora of interventions targeting the pathogenic paradigm of ammonia, inflammation and cerebral haemodynamic dysregulation which are central to the pathogenesis of HE. A greater understanding of the interplay between interorgan ammonia and amino acid metabolism, inflammatory responses and cerebral haemodynamic in ACLF is likely to lead to the development of new therapeutic approaches.
